# Epidemiology and Drug Resistance of Neonatal Bloodstream Infection Pathogens in East China Children’s Medical Center From 2016 to 2020

**DOI:** 10.3389/fmicb.2022.820577

**Published:** 2022-03-10

**Authors:** Xin Zhang, Yang Li, Yunzhen Tao, Yu Ding, Xuejun Shao, Wei Li

**Affiliations:** Department of Clinical Laboratory, Children’s Hospital of Soochow University, Suzhou, China

**Keywords:** newborns, bloodstream infection, resistance, antibacterial drugs, epidemiology

## Abstract

**Introduction:**

To analyze the pathogen distribution and drug resistance of newborns with bloodstream infection (BSI) to help clinicians choose the appropriate empirical antibiotic therapy for clinical infection control.

**Methods:**

A total of 707 neonatal BSI cases were retrospectively analyzed. The bacteria in blood culture-positive samples were cultured, identified, and analyzed for drug sensitivity by routine methods. Statistical software was used to compare and analyze the basic data, pathogenic information, and drug resistance of the main bacteria.

**Results:**

The 5-year average positive rate of neonatal blood culture was 2.50%. The number of specimens submitted for inspection in 2020 significantly decreased. The top five infectious pathogens with the highest proportion were coagulase-negative *Staphylococcus* (67.35%), of which *Staphylococcus epidermidis* had the highest proportion (31.26%), followed by *Escherichia coli* (12.87%), *Klebsiella pneumoniae* (9.05%), *Streptococcus agalactiae* (8.63%), and *Staphylococcus aureus* (3.25%). Gram-positive (G^+^) bacteria were dominant, accounting for 69.45%. The main G^+^ bacteria had a higher rate of resistance to erythromycin and penicillin G. The main Gram-negative (G^–^) bacteria had a high resistance rate to a variety of antibacterial drugs, especially cephalosporin antibiotics. The overall resistance of *K. pneumoniae* was higher than that of *E. coli*. The top two fungi detected were *Candida parapsilosis* and *Candida albicans. C. parapsilosis* did not appear to be resistant to antibiotics, while *C. albicans* was resistant to multiple antibiotics. The type of microbial infection had a statistically significant difference in the positive rate among the age at delivery and wards (*p* < 0.05). There were significant differences in the detection of fungi among these groups (*p* < 0.05). The positive rate of G^+^ bacteria in the term newborns was significantly higher than that in the preterm newborns (*p* < 0.05). Preterm newborns are more susceptible to pneumonia.

**Conclusion:**

G^+^ bacteria are the main pathogens of neonatal BSI. Preterm newborns are more likely to be infected with G^–^ bacteria. *E. coli* and *K. pneumoniae* are the most common G^–^ bacteria, and both have a high resistance rate to a variety of antibacterial drugs. According to the distribution characteristics and drug resistance, it is very important to select antibiotics reasonably.

## Introduction

Infection is the main cause of morbidity in infancy, accounting for 15% of global neonatal deaths (Lucia Hug and You, 2017). Among them, bloodstream infection (BSI) is a common nosocomial type of neonatal death (Yuan et al., 2015). In 2017, the National Bacterial Drug Resistance Monitoring Network reported that 15.2% of bacterial infections in China came from blood samples (Hu et al., 2018). The immune function of newborns is underdeveloped, and resistance is poor. It is very easy to cause sepsis when blood flow infection occurs. The incidence rate of neonatal septicemia among the surviving newborns was 4.5–9.7% (FLeischmann-Struzek et al., 2018). However, due to the clinical use of unilateral blood culture for examination, fewer bacteria, and the use of antibiotics during delivery, blood culture results are often false negative (Klingenberg et al., 2018). The treatment and survival of newborns, especially premature babies, often rely on effective antibiotics, but due to the delay in laboratory tests, empirical medication is often given before the results are available (Puopolo et al., 2018). For neonates, especially premature infants, the use of antibiotics for more than 5 days in infants with negative blood cultures will increase the risk of necrotizing enterocolitis, bronchopulmonary dysplasia, and invasive fungal infections (Ting et al., 2016; Esaiassen et al., 2017). Therefore, the use of big data analysis to explore the results of neonatal drug susceptibility is very important to guide the clinical selection of appropriate antibiotics. At present, there have been research reports on BSI (Spaulding et al., 2019; Johnson et al., 2020; Liu et al., 2020), but due to the influence of factors such as different subjects and regions, the infection characteristics are also different. There are few research papers and comments on the correlation of neonatal BSI in East China. Grasping the distribution characteristics of BSI pathogens in a certain area and performing empirical treatment for the first time are of great significance to saving the lives of newborns. Therefore, a retrospective study of 707 clinical cases of neonatal BSI in East China was performed to understand the composition of pathogenic bacteria and bacterial resistance. The report is as follows.

## Materials and Methods

### General Information

During January 1, 2016, to December 31, 2020, 28,287 blood culture specimens were collected from the Children’s Hospital of Soochow University. A total of 707 newborns with BSI were selected as the research subjects. The inclusion criteria were as follows: (1) newborns; (2) positive blood culture; and (3) increased inflammatory indexes with fever and other blood flow infection symptoms. Among them, 16,040 were male newborns, and 12,244 were female newborns, with a male–female ratio of 1.31:1. According to the age at delivery, the term newborns (11,822 cases) have gestational ages of ≥ 37 weeks, and the preterm newborns (16,465 cases) have gestational ages of < 37 weeks. According to the different admission wards, newborns who have been assessed by the doctor in serious condition will be admitted to the neonatal intensive care unit (NICU) (6,628 cases), and other newborns will be admitted to the general neonatology unit (21,659 cases).

### Instruments and Reagents

The blood culture instrument was purchased from BD (BACTEC FX, United States). The carbon dioxide incubator was purchased from Panasonic (MCO-18AC, Japan). Mass spectrometry was purchased from Bruker (Microflex LT/SH, Germany). The automatic bacterial detection and analysis system was purchased from BioMerieux (VITEK2^®^ compact, France). Drug-sensitive paper was purchased from Oxoid (Basingstoke, Britain). All kinds of culture plates were purchased from Antu (Zhenzhou, China).

### Strain Identification and Drug Sensitivity Test

Blood culture bottles were placed into the instrument for incubation. The positive samples were transferred to the culture plate and incubated at 37°C for 18–24 h (5% CO_2_). The colonies were identified by using a mass spectrometer. The automatic bacterial detection and analysis system and Kirby–Bauer (KB) method were used for the drug sensitivity test. The results were judged according to the latest standards of the Clinical Laboratory Standardization Association (Clinical and Laboratory Standards Institute, 2020). Extended-spectrum β-lactamases (ESBLs) were determined by the automatic bacterial detection and analysis system. The judgment results were obtained according to its own expert system. The quality control strains were *Escherichia coli* (*ATCC 25922*), *Pseudomonas aeruginosa* (*ATCC 27853*), *Staphylococcus aureus* (*ATCC 25923* and *ATCC 29213*), *Enterococcus faecalis* (*ATCC 29212*), and *Streptococcus pneumoniae* (49619), which were purchased from the clinical testing center of the National Health Commission.

### Statistical Analysis

SPSS 20.0 and WHONET 5.6 were used to analyze data. The counting data were expressed as the number of cases (n) and rate (%). The χ^2^-test was used in univariate analysis. The comparison between groups was carried out by the χ^2^-test, with *p* < 0.05 as the difference, which was statistically significant.

## Results

### Annual Distribution of Pathogenic Bacteria [*n* (%)]

**FIGURE 1:**
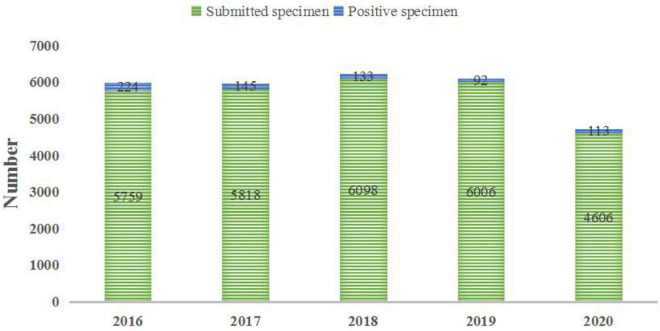
Analysis of bacterial detection in 2016–2020.

The positive rates in the 5 years from 2016 to 2020 were 3.89, 2.49, 2.18, 1.53, and 2.45%, respectively. In 707 cases of neonatal BSI, 491 strains of Gram-positive (G^+^) bacteria were isolated, accounting for 69.45%, and among them were *Staphylococcus epidermidis* (31.26%), *Streptococcus agalactiae* (8.63%), and *Staphylococcus hominis* (8.20%). Strains of Gram-negative (G^–^) bacteria (182) were isolated, accounting for 25.74%, and among them were *E. coli* (12.87%) and *Klebsiella pneumoniae* (9.05%). Strains of fungi (Akbarian-Rad et al., 2020) were isolated, accounting for 4.24% (see Figure 1 and Table 1 for details).

**TABLE 1:** Annual distribution of pathogenic bacteria [*n* (%)].

Type	2016	2017	2018	2019	2020	Total	Percentage (%)
**Gram-positive bacteria**	**154**	**104**	**86**	**62**	**85**	**491**	**69.45**
*Staphylococcus epidermidis*	83	39	33	30	36	221	31.26
*Staphylococcus hominis*	27	9	10	5	7	58	8.2
*Staphylococcus capitis*	7	16	8	6	8	45	6.36
*Staphylococcus haemolyticus*	8	4	4	2	0	18	2.55
*Staphylococcus warneri*	1	1	5	0	4	11	1.56
*Staphylococcus caprae*	0	0	0	0	3	3	0.42
*Staphylococcus lugdunensis*	0	0	1	0	2	3	0.42
*Streptococcus agalactiae*	11	12	12	5	21	61	8.63
*Staphylococcus aureus*	6	8	0	7	2	23	3.25
*Listeria monocytogenes*	5	0	9	3	0	17	2.4
*Enterococcus faecium*	2	4	2	2	2	12	1.70
*Enterococcus faecalis*	1	0	0	0	0	1	0.14
*Streptococcus pallidus*	2	6	1	0	0	9	1.27
Other *Streptococcus*	1	5	1	2	0	9	1.27
**Gram-negative bacteria**	**56**	**37**	**41**	**26**	**22**	**182**	**25.74**
*Escherichia coli*	26	12	24	15	14	91	12.87
*Klebsiella pneumoniae*	25	15	9	7	8	64	9.05
*Enterobacter cloacae*	2	4	1	2	0	9	1.27
*Enterobacter aerogenes*	0	3	1	0	0	4	0.57
*Enterobacter asheri*	0	0	0	2	0	2	0.28
*Klebsiella oxytoca*	0	0	2	0	0	2	0.28
*Citrobacter Klebsiella*	0	0	2	0	0	2	0.28
*Serratia marcescens*	0	1	0	0	0	1	0.14
*Salmonella* Typhimurium	0	0	1	0	0	1	0.14
*Pseudomonas stephensi*	0	0	1	0	0	1	0.14
*Acinetobacter baumannii*	1	0	0	0	0	1	0.14
*Elizabetha meningealis*	1	0	0	0	0	1	0.14
*Acinetobacter yoelii*	0	1	0	0	0	1	0.14
Hospital *Acinetobacter*	1	0	0	0	0	1	0.14
*Acinetobacter pittii*	0	1	0	0	0	1	0.14
**Fungi**	**14**	**4**	**6**	**3**	**3**	**30**	**4.24**
*Candida parapsilosis*	11	1	4	0	0	16	2.26
*Candida albicans*	3	3	2	3	1	12	1.7
*Candida jiyemeng*	0	0	0	0	2	2	0.28
**Mix-infection**	**0**	**0**	**0**	**1**	**3**	**4**	**0.57**
**Total**	**224**	**145**	**133**	**92**	**113**	**707**	**100**

### Resistance Rate of the Main G^+^ Bacteria to Common Antibiotics (%)

Coagulase-negative *Staphylococcus* (CNS), *S. agalactiae*, *S. aureus*, and *Enterococcus* are the main G^+^ bacteria. Table 2 shows that the above bacteria are resistant to various antibiotics to varying degrees. They have a higher rate of resistance to erythromycin. *Staphylococcus* has over 80% resistance to penicillin G, and *S. agalactiae* is 100% sensitive to penicillin G (see Table 2 for details).

**TABLE 2:** Resistance rate of the main G^+^ bacteria to common antibiotics (%).

Antibiotics	*Staphylococcus epidermidis* (*n* = 205)	*Staphylococcus haemolyticus* (*n* = 18)	*Streptococcus agalactiae* (*n* = 45)	*Staphylococcus aureus* (*n* = 20)	*Enterococcus faecium* (*n* = 11)
Oxacillin	68.78	88.89	−	35.00	–
Sulfamethoxazole	34.15	33.33	−	5.00	–
Erythromycin	72.20	94.44	91.11	50.00	–
Ciprofloxacin	25.85	77.78	−	0.00	–
Quinuptin/Dafopudin	0.00	0.00	6.67	0.00	0.00
Linezolid	0.00	0.00	0.00	0.00	0.00
Rifampicin	8.78	22.22	−	0.00	100
Clindamycin	28.78	44.44	80.00	50.00	–
Moxifloxacin	3.41	55.56	−	0.00	–
Penicillin G	89.76	88.89	0.00	85.00	100
Gentamicin	14.15	72.22	−	5.00	–
Tetracycline	13.17	38.89	73.33	5.00	–
Tigecycline	0.00	72.22	−	0.00	0.00
Cefoxitin	38.89	88.89	−	65.00	–
Vancomycin	0.00	0.00	0.00	0.00	0.00
Levofloxacin	27.80	77.78	33.33	0.00	81.82
Minocycline	−	−	−	−	72.72

*“−”: This means it is not detected.*

### Resistance Rate of the Main G^–^ Bacteria to Common Antibiotics (%)

**FIGURE 2:**
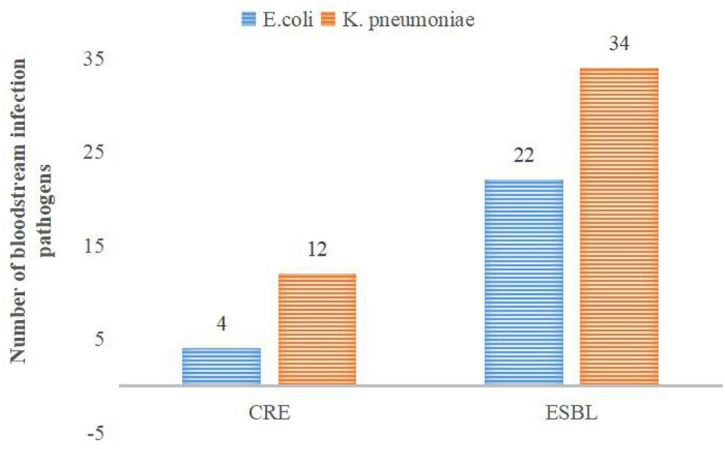
Distribution of the main drug-resistant bacteria.

*E. coli* and *K. pneumoniae* are the main G^–^ bacteria. *K. pneumoniae* produces ESBLs, accounting for up to 55.73%. The overall resistance of *K. pneumoniae* is higher than that of *E. coli* (see Table 3 for details). Subsequently, the multidrug resistances of *K. pneumoniae* and *E. coli* were analyzed. The results are shown in Figure 2.

**TABLE 3:** Resistance rate of the main G^–^ bacteria to common antibiotics (%).

Antibiotics	*Escherichia coli* (*n* = 75)	*Klebsiella pneumoniae* (*n* = 56)
ESBLs	27.16	55.73
Ampicillin	73.33	100
Ampicillin/Sulbactam	32	83.93
Aztreonam	17.33	21.43
Amikacin	0	1.96
Sulfamethoxazole	50.67	55.36
Ciprofloxacin	33.33	10.71
Levofloxacin	32	0
Piperacillin	41.93	83.33
Piperacillin/Tazobactam	4.50	5.36
Gentamicin	30.67	7.14
Tobramycin	9.33	10.71
Cefotetan	4.05	23.21
Ceftazidime	13.33	55.36
Cefatriaxone	33.33	75
Cefepime	8.21	50
Cefazolin	33.33	80.36
Cefoxitin	8.21	35.71
Cefazoxime	18.67	50
Cefuroxime	25	76.78
Cefotaxime	40.90	69.23
Cefoperazone/Sulbactam	8.21	30.36
Imipenem	7.24	21.43
Meropenem	6.81	32

### Comparison of Positive Rates of Bloodstream Infection in Newborns of Different Groups [*n* (%)]

The type of microbial infection had a statistically significant difference in the positive rate among the age at delivery and the ward (*p* < 0.05). There were significant differences in the detection of fungi among these groups (*p* < 0.05). Details are shown in Table 4.

**TABLE 4:** Comparison of the positive rate of BSI in newborns of different groups (*n*).

Index		Total	Positive number	G^+^ bacteria	G^–^ bacteria	fungi	Mix-infection
Gender	Male	16,040	404	283	110	10	1
	Female	12,244	303	208	72	20	3
	χ^2^	–	0.055	0.175	1.038	6.685	1.639
	*p*	–	0.814	0.676	0.308	**0.01**	0.201
	OR	–	1.018	1.039	1.167	0.381	0.254
	95% CI	–	0.876–1.184	0.868–1.245	0.866–1.573	0.178–0.815	0.026–2.446
Age at delivery	Term newborns	11,822	316	249	63	1	3
	Preterm newborns	16,465	391	242	119	29	1
	χ^2^	–	2.512	16.342	3.879	18.261	1.813
	*p*	–	0.113	**<0.001**	**0.049**	**<0.001**	0.178
	OR	–	1.129	1.442	0.736	0.048	4.179
	95% CI	–	0.972–1.312	1.207–1.724	0.542–1.000	0.007–0.352	0.435–40.180
Ward	NICU	6,628	255	135	89	30	1
	Neonatology	21,659	452	356	93	0	3
	χ^2^	–	64.54	4.599	66.234	98.138	0.005
	*p*	–	**<0.001**	**0.032**	**<0.001**	**<0.001**	0.941
	OR	–	1.877	1.244	3.156	1.005	1.089
	95% CI	–	1.606–2.194	1.019–1.520	2.357–4.226	1.003–1.006	0.113–10.474

*OR, the odds ratio; 95% CI, the 95% confidence interval.*

### Clinical Diagnoses

Among the clinical diagnoses, pneumonia, jaundice, purulent meningitis, newborn enteritis, and newborn intestinal obstruction accounted for the top five, of which pneumonia accounted for 53.18%, followed by jaundice, accounting for 24.75% (see more in Table 5). Pathogens detected in the different groups are further analyzed in Table 6.

**TABLE 5:** Comparison of clinical diagnoses of different groups (*n*).

Index		Pneumonia	Jaundice	Purulent meningitis	Enteritis	Intestinal obstruction	Respiratory distress syndrome	Urinary tract infection	Vomiting	Hyperbilirubinemia	Other infections	Other symptoms
Gender	Male	218	102	17	11	9	4	8	4	6	5	20
	Female	158	73	21	10	8	13	3	5	0	7	5
	χ^2^	0.25	0.178	2.222	0.165	0.098	7.629	1.15	0.552	4.581	1.107	5.528
	*p*	0.617	0.673	0.136	0.685	0.754	0.006	0.284	0.458	**0.032**	0.293	0.019
	OR	1.054	1.067	0.618	0.837	0.859	0.235	2.036	0.611	1	0.545	3.056
	95% CI	0.858–1.295	0.789–1.442	0.326–1.171	0.356–1.973	0.331–2.226	0.077–0.720	0.540–7.676	0.164–2.274	1.000–1.001	0.173–1.718	1.147–8.145
Age at delivery	Term newborns	144	106	19	7	9	4	3	3	2	8	11
	Preterm newborns	232	69	19	14	8	13	8	6	4	4	14
	χ^2^	1.914	25.525	1.054	0.618	0.869	2.332	0.954	0.265	0.177	3.053	0.050
	*p*	0.167	**<0.001**	0.305	0.432	0.351	0.127	0.329	0.607	0.674	0.081	0.823
	OR	0.863	2.150	1.393	0.696	1.567	0.428	0.522	0.696	0.696	2.787	1.094
	95% CI	0.700–1.064	1.586–2.914	0.737–2.633	0.281–1.725	0.605–4.063	0.140–1.314	0.138–1.969	0.174–2.785	0.128–3.802	0.839–9.256	0.497–2.411
Ward	NICU	177	7	16	8	10	16	1	4	6	2	8
	Neonatology	199	168	22	13	7	1	10	5	0	10	17
	χ^2^	118.731	37.059	7.396	2.519	11.876	47.373	1.261	2.216	19.611	0.306	1.024
	*P*	**<0.001**	**<0.001**	**0.007**	0.112	**0.001**	**<0.001**	0.261	0.137	** < 0.001**	0.58	0.132
	OR	2.959	0.135	2.38	2.012	4.674	52.409	0.327	2.615	1.001	0.653	1.538
	95% CI	2.412–3.630	0.063–0.288	1.249–4.534	0.834–4.857	1.778–12.283	6.949–395.262	0.042–2.552	0.702–9.742	1.000–1.002	0.143–2.983	0.664–3.566
Infection	G + bacteria	246	147	27	13	11	5	4	9	0	10	19
	G^–^ bacteria	109	28	10	8	5	1	7	0	6	2	6
	fungi	17	0	1	0	1	11	0	0	0	0	0
	Mix-Infection	4	0	0	0	0	0	0	0	0	0	0
Total		376	175	38	21	17	17	11	9	6	12	25

*OR, the odds ratio; 95% CI, the 95% confidence interval.*

**TABLE 6:** Analysis of bacterial species of different groups (*n*).

Group		G^+^ bacteria							G^–^ bacteria					Fungi	Mix-infection	Total
		*Staphylococcus epidermidis*	Other CNS	*Streptococcus agalactiae*	*Staphylococcus aureus*	*Listeria monocytogenes*	*Enterococcus*	Other *Streptococcus*	*Escherichia coli*	*Klebsiella pneumoniae*	*Enterobacter cloacae*	*Enterobacter aerogenes*	Other G^–^ bacteria			
Gender	Male	123	71	43	11	9	8	10	62	38	8	3	7	10	1	404
	Female	98	67	18	12	8	5	8	29	26	1	1	7	20	3	303
Age at delivery	Term newborns	125	60	38	11	5	5	5	43	10	7	1	2	1	3	316
	Preterm newborns	96	78	23	12	12	8	13	48	54	2	3	12	29	1	391
Ward	NICU	35	38	27	7	15	9	4	23	53	5	3	5	30	1	255
	Neonatology	186	100	34	16	2	4	14	68	11	4	1	9	0	3	452
Clinical diagnoses	Pneumonia	98	72	37	9	13	7	10	42	51	4	3	9	17	4	376
	Jaundice	91	46	2	3	0	1	4	20	6	0	1	1	0	0	175
	Purulent meningitis	2	2	15	4	4	0	0	8	0	0	0	2	1	0	38
	Enteritis	7	2	0	2	0	1	1	1	5	0	0	2	0	0	21
	Intestinal Obstruction	4	2	2	1	0	2	0	2	2	1	0	0	1	0	17
	Respiratory distress syndrome	1	2	1	0	0	0	1	1	0	0	0	0	11	0	17
	Urinary tract infection	2	2	0	0	0	0	0	7	0	0	0	0	0	0	11
	Vomiting	3	3	0	0	0	2	1	0	0	0	0	0	0	0	9
	Hyperbilirubinemia	0	0	0	0	0	0	0	6	0	0	0	0	0	0	6
	Other infections	4	2	0	4	0	0	0	0	0	2	0	0	0	0	12
	Other symptoms	9	5	4	0	0	0	1	4	0	2	0	0	0	0	25
Total		221	138	61	23	17	13	18	91	64	9	4	14	30	4	707

## Discussion

BSI has a high incidence and accounts for a high proportion of nosocomial infections, and its incidence has been increasing in recent years. It is complicated and poorly effective. According to reports, the in-hospital mortality rate of sepsis is as high as 30–60% (Bouza et al., 2015), exceeding the sum of mortalities due to acquired immune deficiency syndrome, breast cancer, and prostate cancer. For every hour of delay in treatment, the patient’s mortality rate will increase by 7.6% (Kumar et al., 2006). International guidelines recommend that effective antibiotics should be used intravenously within 1 h after sepsis is diagnosed (Dellinger et al., 2013).

In this study, the 5-year average positive rate of neonatal blood culture was 2.50%. As shown in Figure 1, the positive rates of blood culture from 2016 to 2020 were 3.89, 2.49, 2.18, 1.53, and 2.45%, respectively. Even during the COVID-19 pandemic, the total number of specimens submitted for clinical examination decreased, but it did not affect the detection of positive specimens. The 5-year average distribution of pathogens was mainly G^+^ bacteria (69.45%), which is consistent with previous similar research results (Jing and Big, 2019). However, a survey of pathogens of neonatal sepsis from Nigeria showed that the infection was mainly G^–^ bacteria (Pius et al., 2016). The above differences suggest that the distribution of common pathogens of neonatal BSI may have differences in research time, research locations, or research objects, which only represent the situation of the research institution at a certain time. CNS is the main pathogen of neonatal blood flow infection in the hospital, accounting for 50.77%, but the CNS isolated from a single blood culture may also be contaminated (García-Gudiño et al., 2017). Therefore, blood collection personnel should pay special attention to aseptic operation and hand hygiene. Nevertheless, the positive rate of CNS is still very high in neonatal blood culture, as reported by Siti et al. (2020). The detection of *Streptococcus* is very important for perinatal pregnant women, especially those with premature rupture of membranes. Previous studies have found that the colonization rate of *S. agalactiae* in pregnant women is 19% (Lixiang et al., 2018). *S. agalactiae* was the second most susceptible G^+^ bacteria to neonatal BSI in this study, accounting for 8.63%, which is consistent with the results reported in other literature (Lili et al., 2017). It is worth noting that the ratio of *S. agalactiae* among the detected G^+^ bacteria was quite different in 2020 (∼25%) compared with other years (7–14%). Recent studies have shown that *S. agalactiae* is closely related to COVID-19 (Soto et al., 2021; Xiong et al., 2021). The high ratio of *S. agalactiae* among the detected G^+^ bacteria in 2020 may be related to the environment of COVID-19 infection, but further studies are needed. There are relatively few cases of neonatal BSI caused by *S. aureus*. Only 23 strains were found in this study, of which 8 strains were methicillin-resistant *S. aureus* (MRSA). The positive rate of MRSA is significantly lower in neonates than that in older children (Seas et al., 2018; Fang et al., 2020). The drug sensitivity results showed that the drug resistance rates of the main G^+^ bacteria to erythromycin and penicillin G were high, while they were 100% sensitive to vancomycin and linezolid. The resistance rates of CNS represented by *S. epidermidis* to penicillin G, erythromycin, and oxacillin were 89.76, 72.20, and 68.78%, respectively, and it is 100% sensitive to quinuptin/dafopudin, linezolid, and vancomycin. The resistance rate to moxifloxacin was 3.41% based on the overall antimicrobial susceptibility testing profile. It should be noted that the resistance rates of *Staphylococcus epidermidis* to moxifloxacin from 2016 to 2020 were 3.7, 7.69, 2.94%, 0, and 0, respectively. This suggests that in the case of poor efficacy of conventional drugs, moxifloxacin can be selected according to the situation of patients. In this study, *Staphylococcus haemolyticus* had high resistance to most antibiotics. There were 16 strains of methicillin-resistant *S. haemolyticus.* According to Takeuchi et al. (2005) *S. haemolyticus* has the maximum level of antimicrobial resistance among all CNS species. In recent years, linezolid-resistant *S. haemolyticus* has been reported (Rajan et al., 2017; Ahmed et al., 2019), and no quinuptin/dafopudin-, linezolid-, or vancomycin-resistant *S. haemolyticus* were reported in the present study. *S. agalactiae* has different degrees of resistance to common antibiotics, and the rate of resistance to erythromycin is the highest, up to 91.11%. However, it is 100% sensitive to penicillin G, so penicillin G is also the first choice for the treatment of neonatal *S. agalactiae* infection. The resistance rate of *S. aureus* to penicillin G was as high as 85%, but to macrolide antibiotics represented by erythromycin, it was 50%. Therefore, macrolides have been widely used in neonatal and perinatal diseases in recent years (Wang et al., 2015). *Enterococcus faecium* has a high resistance rate to a variety of antibiotics, such as 100% resistance to rifampicin and penicillin G, 81.82% resistance to levofloxacin, and 72.72% resistance to minocycline. However, it was 100% sensitive to tigecycline and linezolid. There were no vancomycin-resistant *E. faecium* strains. See Supplementary Material 1 for more antimicrobial susceptibility testing results of pathogen.

In the present research, 182 G^–^ bacterial strains were detected, accounting for 25.74%. The proportion of the positive rate of G^–^ bacteria was lower than that in previous similar literature reports (Pius et al., 2016; Akbarian-Rad et al., 2020). This may be related to the high positive rate of CNS (accounting for 50.77%) in the study. The main G^–^ bacteria in neonatal BSI were *E. coli* and *K. pneumoniae*, which is consistent with the study by other developing countries (Dat et al., 2017). The detected proportions of *E. coli* and *K. pneumoniae* were 12.87 and 9.05%, respectively. Interestingly, there was a little difference in the rate of *E. coli* and *K. pneumoniae* among the detected G^–^ bacteria from 2016 to 2017. However, after 2018, the proportion of *E. coli* significantly increased and was nearly twice that of *K. pneumoniae*. Nevertheless, due to the small number of positive specimens every year, it is necessary to continue to track the changes in detected bacteria in follow-up studies. Both *K. pneumoniae* and *E. coli* have high resistance to ampicillin, trimethoprim, and cefotaxime, which is similar to the results of Michael et al. (2017). However, the resistance rate to piperacillin/tazobactam, ciprofloxacin, aztreonam, and so on is low. As shown in Table 3, *K. pneumoniae* was 100% resistant to ampicillin, and the resistance rate to ampicillin/sulbactam reached 83.93%. The resistance rate to second- and third-generation cephalosporins is very high, and the resistance rate to some antibiotics is as high as 50%. The resistance rate of *E. coli* to cefuroxime was 25%, the resistance rate to ceftriaxone was 33.33%, and the resistance rate to fourth-generation cefotaxime was as high as 40.90%. The above results indicate that the resistance rate of *K. pneumoniae* to multiple antibacterial drugs is significantly higher than that of *E. coli*. The high resistance of bacteria to a variety of cephalosporin antibiotics indicates that the proportion of bacteria producing ESBLs is relatively high. In this study, the positive rates of ESBLs-producing *K. pneumoniae* and *E. coli* were 55.73 and 27.16%, respectively, which are lower than the results reported in previous studies (Wang et al., 2020). In recent years, the clinical isolation rate of carbapenem-resistant *Enterobacter* (CRE), especially carbapenem-resistant *K. pneumoniae* (CRKP), has increased (Stein et al., 2019). According to data from the China Antibiotic Resistance Surveillance Network, the isolation rate of CRKP among Chinese children rose from 3.0 to 20.9% from 2005 to 2017, which was significantly higher than that of adults (Wang et al., 2020). As shown in Figure 2, there were 12 strains of CRKP and 4 strains of carbapenem-resistant *E. coli*, which accounted for 18.75 and 4.40% of their respective strains. Carbapenem-resistant strains are resistant to most antibacterial drugs, and clinical treatment measures are limited. Therefore, it is difficult to control infection, and the mortality rate is high (Gu et al., 2018). Special attention should be given to the abovementioned carbapenem-resistant strains to actively prevent infection.

In recent years, BSI caused by fungi has increased significantly, and *Candida* is the most common fungi (Dilhari et al., 2016). Previous studies reported that the neonatal BSI caused by fungi was mainly by *Candida albicans* (Ting et al., 2018), which is somewhat different from the results of this study. In this study, a total of 30 strains of fungi were detected, including 16 strains of *Candida parapsilosis* and 12 strains of *C. albicans*. This may be related to the low number of fungi detected in this study. *C. parapsilosis* is 100% sensitive to 5-fluorocytosine, voriconazole, fluconazol, itraconazole, and amphotericin B, which is also consistent with the conclusion reported by Van Asbeck et al. (2009). Drug resistance was concentrated on *C. albicans*, and two strains of resistant *C. albicans* were obtained. One strain was only resistant to 5-fluorocytosine, and the other was only sensitive to amphotericin B. The low resistance rate to amphotericin B may be related to the greater side effects of the drug.

The differences in pathogen detection between genders, age at delivery, and ward were further analyzed. In Table 4, fungi were a risk factor for BSI in female newborns. The positive rate of G^+^ bacteria in term newborns was 2.11% (249/11,822), which was higher than the 1.47% (242/11,465) of preterm newborns, while the positive rate of G^–^ bacteria was the opposite. The above differences were statistically significant (*p* < 0.05). These results are consistent with the finding that preterm newborns are more susceptible to G^–^ bacteria (Zhang, 2020). The study also found that 30 patients with fungal infections were all preterm newborns, and the difference in fungal detection between preterm newborns and term newborns was statistically significant (χ^2^ = 18.261, *p* < 0.001). Studies have shown that the immune function of preterm newborns is relatively weak, and they are more prone to fungal infections (Manzoni et al., 2015). Most of the newborns admitted to the NICU have more serious underlying diseases, may undergo more invasive procedures, and have a significantly increased risk of infection. Statistics found that the positive rates of G^±^ bacteria and fungi in newborns in the NICU were significantly higher than those in neonatology, and the difference was statistically significant (*p* < 0.05). Therefore, the ICU should pay special attention to aseptic operation and hand hygiene and reduce cross-infection among newborns. In the main clinical diagnosis, the top five diseases were pneumonia (53.18%), jaundice (24.75%), purulent meningitis (5.37%), and neonatal enteritis (2.97%), the intestinal obstruction and respiratory distress syndrome ranked fifth (2.40%). Statistical analysis of the prevalence of pneumonia and jaundice found that compared with term newborns, preterm newborns are more susceptible to pneumonia and have a lower risk of jaundice, which is consistent with the results reported by Chen et al. (2017). The above results may be related to premature newborns due to immature lung development and more mechanical ventilation, so they are prone to respiratory tract infections which induce severe pneumonia. The study also found that the positive rate of pneumonia among newborns in the NICU was 2.67%, which was significantly higher than that of newborns in neonatology (0.92%), and the difference in detection was statistically significant (χ^2^ = 118.731, *p* < 0.001). Among the 255 patients with BSI in the NICU, there were 181 preterm newborns, of which 141 were clinically diagnosed with pneumonia, accounting for 77.91%. Therefore, it is necessary to pay special attention to the correlation between bloodstream infection and pneumonia in premature infants to be vigilant and take preventive measures as early as possible.

Subsequently, the types and quantities of bacteria among different genders, age at delivery, wards, and clinical diagnoses were determined. As shown in Table 6, many clinical diagnoses were closely related to the detection of G^+^ bacteria, except urinary tract infection and hyperbilirubinemia. It is worth noting that 17 strains of *Listeria monocytogenes* were detected in the present study. Among them, 6 strains of *L. monocytogenes* entered the cerebrospinal fluid and caused meningitis. *L. monocytogenes* is a pathogen of sepsis and meningitis in children. A study on *L. monocytogenes* infection shows that infection in children is common in newborns, especially in preterm newborns, which easily causes suppurative meningitis (Cai et al., 2020). The detection of *L. monocytogenes* is closely related to preterm newborns admitted to the NICU with purulent meningitis and pneumonia in this study. Therefore, the neonatal ward should attach great importance to the spread of bacterial infection and do a good job in the prevention and control measures of cross infection.

In summary, regular monitoring of bacterial resistance and understanding of changes in pathogen spectrum and antimicrobial resistance patterns will help clinicians use drugs rationally and better prevent and control the occurrence of infectious diseases. Corresponding wards should pay attention to the inspection rate of blood cultures, consider the trend of drug resistance in the hospital, adjust medications, and reduce infection mortality. However, there are some limitations in this study. The overall positive rate and drug resistance of pathogens in the last 5 years were analyzed. There is a lack of further exploration of the resistance changes of various antimicrobials. It is also significant to analyze the change in the positive rate and drug resistance rate of each pathogen during the epidemic period of COVID-19. Therefore, we will pay more attention to the above limitations in a follow-up study.

## Data Availability Statement

The raw data supporting the conclusions of this article will be made available by the authors, without undue reservation.

## Ethics Statement

The studies involving human participants were reviewed and approved by the Medical Ethics Committee of the Children’s Hospital of Soochow University (ethics batch number: 2021CS158). Written informed consent to participate in this study was provided by the participants’ legal guardian/next of kin. Written informed consent was obtained from the individual(s) for the publication of any potentially identifiable images or data included in this article.

## Author Contributions

XZ and WL conceived the study and designed the experiments. YL and XS provided financial support. XS, YD, and YT collected, analyzed the data, and interpreted the results. XZ drafted the manuscript. All authors critically revised the manuscript for intellectual content and read and approved the final manuscript.

## Author Disclaimer

The views, opinions, assumptions, or any other information set out in this article are solely those of the authors and should not be attributed to the funders or any other person connected with the funders.

## Conflict of Interest

The authors declare that the research was conducted in the absence of any commercial or financial relationships that could be construed as a potential conflict of interest.

## Publisher’s Note

All claims expressed in this article are solely those of the authors and do not necessarily represent those of their affiliated organizations, or those of the publisher, the editors and the reviewers. Any product that may be evaluated in this article, or claim that may be made by its manufacturer, is not guaranteed or endorsed by the publisher.
